# Endoscopic submucosal dissection using the water pressure method for superficial esophageal cancer within a cervical pseudodiverticulum

**DOI:** 10.1055/a-2840-7806

**Published:** 2026-04-15

**Authors:** Hideomi Tomida, Kazuhiro Tange, Yasunori Yamamoto, Eiji Takeshita, Yoshiou Ikeda, Yoichi Hiasa

**Affiliations:** 1 89456 Endoscopy Center, Ehime University Hospital, Toon, Japan; 2 38050 Department of Inflammatory Bowel Diseases and Therapeutics, Ehime University Graduate School of Medicine, Toon, Japan; 3 38050 Department of Gastroenterology and Metabology, Ehime University Graduate School of Medicine, Toon, Japan

The water pressure method (WPM), an underwater technique utilizing normal saline immersion and an active water stream to visualize the submucosa, was developed to enhance the safety of endoscopic submucosal dissection (ESD) for technically challenging duodenal lesions 1 2 . Its efficacy has also been demonstrated in the hypopharynx and colon 3 4 . We report the successful application of WPM-assisted ESD for a rare superficial esophageal cancer arising within a cervical pseudodiverticulum ( Video 1 ).

Endoscopic submucosal dissection using the water pressure method for superficial esophageal cancer within a cervical pseudodiverticulum.Video 1

**Fig. 1:**
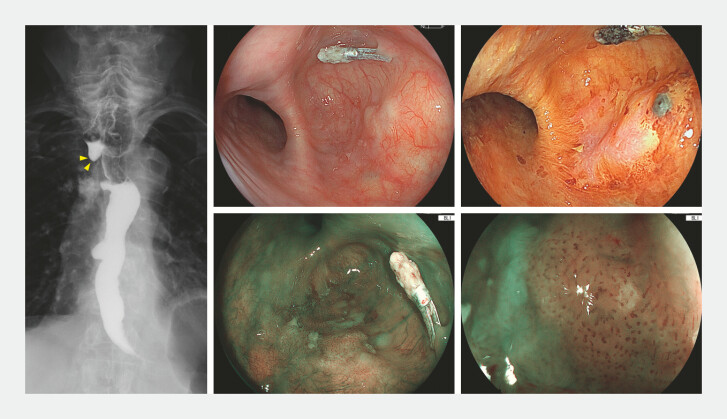
Endoscopy showed a pseudodiverticulum in the cervical esophagus (yellow arrowhead) containing a 15-mm type 0–Ib tumor. The depth of tumor invasion was clinically diagnosed as EP/LPM.

A 76-year-old man was referred to our hospital for the treatment of superficial esophageal cancer located within a cervical pseudodiverticulum ( Fig. 1 ). An open surgical diverticulectomy with clavicular head resection had previously been attempted but was aborted due to anatomical inaccessibility. Because further surgery carried an unavoidable risk of recurrent laryngeal nerve paralysis and severe dysphagia, an endoscopic approach was indicated. However, conventional gas-insufflation ESD posed a substantial perforation risk due to the absence of a muscularis propria in the pseudodiverticulum; therefore, we opted for WPM-assisted ESD under general anesthesia.

**Fig. 2:**
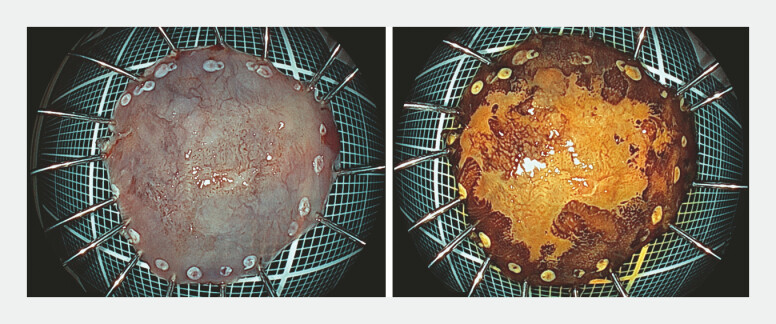
Histopathology of the resected specimen: 15 × 14 mm, type 0–IIb, squamous cell carcinoma, pT1a-LPM, Ly0, V0, pHM0 and pVM0.

**Fig. 3:**
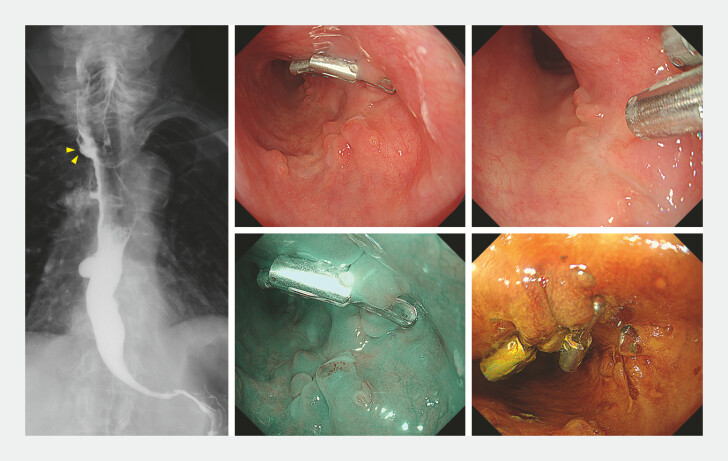
Follow-up endoscopy at 2 months: the pseudodiverticulum had shrunk (yellow arrowhead) and no residual tumor was observed.

Following luminal filling with normal saline, mucosal incision was initiated. Submucosal dissection was advanced from the oral side using the WPM, carefully preserving the submucosal layer. En bloc resection was completed in 83 minutes. The mucosal defect was completely closed with through-the-scope clips, and the procedure performed without adverse events ( Fig. 2 ). Histopathological examination revealed a 15 × 14 mm type 0-IIb squamous cell carcinoma confined to the lamina propria mucosae, with negative margins and no lymphovascular invasion ( Fig. 2 ). Follow-up endoscopy at 2 months showed no residual tumor and a reduction in size of the pseudodiverticulum ( Fig. 3 ).

To the best of our knowledge, this is the first reported case of successful ESD for esophageal cancer arising within a pseudodiverticulum. WPM-assisted ESD may represent a safe and effective therapeutic option for such highly challenging cases.

Endoscopy_UCTN_Code_TTT_1AO_2AG_3AD
